# Mammalian predator and prey responses to recreation and land use across multiple scales provide limited support for the human shield hypothesis

**DOI:** 10.1002/ece3.10464

**Published:** 2023-09-14

**Authors:** Alys Granados, Catherine Sun, Jason T. Fisher, Andrew Ladle, Kimberly Dawe, Christopher Beirne, Mark S. Boyce, Emily Chow, Nicole Heim, Mitchell Fennell, Joanna Klees van Bommel, Robin Naidoo, Michael Procko, Frances E. C. Stewart, A. Cole Burton

**Affiliations:** ^1^ Department of Forest Resources Management University of British Columbia Vancouver British Columbia Canada; ^2^ Institute for Resources, Environment and Sustainability University of British Columbia Vancouver British Columbia Canada; ^3^ School of Environmental Studies University of Victoria Victoria British Columbia Canada; ^4^ Quest University Canada Squamish British Columbia Canada; ^5^ Department of Biological Sciences University of Alberta Edmonton Alberta Canada; ^6^ British Columbia Ministry of Forests, Lands, Natural Resource Operations and Rural Development Cranbrook British Columbia Canada; ^7^ Ktunaxa Nation Government Cranbrook British Columbia Canada; ^8^ World Wildlife Fund‐US Washington DC USA; ^9^ Department of Biology Wilfrid Laurier University Waterloo Ontario Canada; ^10^ Department of Forest Resources Management and Biodiversity Research Centre University of British Columbia Vancouver British Columbia Canada

**Keywords:** Bayesian inference, camera trap, mammal conservation, protected area, recreation

## Abstract

Outdoor recreation is widespread, with uncertain effects on wildlife. The human shield hypothesis (HSH) suggests that recreation could have differential effects on predators and prey, with predator avoidance of humans creating a spatial refuge ‘shielding’ prey from people. The generality of the HSH remains to be tested across larger scales, wherein human shielding may prove generalizable, or diminish with variability in ecological contexts. We combined data from 446 camera traps and 79,279 sampling days across 10 landscapes spanning 15,840 km^2^ in western Canada. We used hierarchical models to quantify the influence of recreation and landscape disturbance (roads, logging) on ungulate prey (moose, mule deer and elk) and carnivore (wolf, grizzly bear, cougar and black bear) site use. We found limited support for the HSH and strong responses to recreation at local but not larger spatial scales. Only mule deer showed positive but weak landscape‐level responses to recreation. Elk were positively associated with local recreation while moose and mule deer responses were negative, contrary to HSH predictions. Mule deer showed a more complex interaction between recreation and land‐use disturbance, with more negative responses to recreation at lower road density or higher logged areas. Contrary to HSH predictions, carnivores did not avoid recreation and grizzly bear site use was positively associated. We also tested the effects of roads and logging on temporal activity overlap between mule deer and recreation, expecting deer to minimize interaction with humans by partitioning time in areas subject to more habitat disturbance. However, temporal overlap between people and deer increased with road density. Our findings highlight the complex ecological patterns that emerge at macroecological scales. There is a need for expanded monitoring of human and wildlife use of recreation areas, particularly multi‐scale and ‐species approaches to studying the interacting effects of recreation and land‐use change on wildlife.

## Graphical abstract

Outdoor recreation is widespread, with uncertain effects on wildlife. We used 446 camera traps in Western Canada to measure mammal predator and prey multi‐scale responses to recreation, according to the Human Shield Hypothesis. Responses were mostly local and depended on co‐occuring disturbances and, contrary to predictions under the hypothesis, most ungulates avoided recreation while carnivores did not.

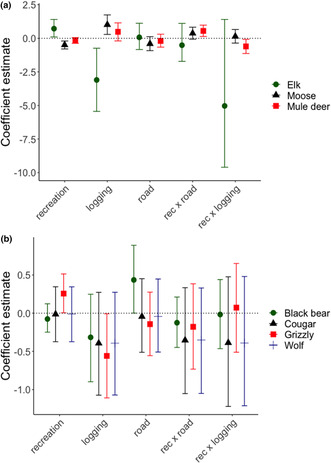

## 1 INTRODUCTION

Outdoor recreation is widespread and increasing across a range of ecosystems that many wildlife species inhabit (Dertien et al., 2021; Larson et al., 2016). Even within protected areas (PAs), human disturbance from recreation and other activities is prevalent and growing (Balmford et al., 2015; Watson et al., 2014). Wildlife must also navigate a matrix of urban, industrial and wilderness areas with varying degrees of landscape disturbance and human activity. The impacts of recreation on wildlife have been documented in single‐site studies, illustrating diverse consequences for animal habitat use and activity patterns (Ladle et al., 2018; Lewis et al., 2021; Naidoo & Burton, 2020; Procko et al., 2022) and the effects of recreation on wildlife are expected to be globally widespread yet context‐dependent (Larson et al., 2016). This raises questions about our ability to extrapolate from individual sites to make generalizable predictions about impacts on wildlife (Peters, 1991). Predictable patterns at macroecological scales (McGill, 2019)—if they exist—would inform management actions in the many areas that lack adequate monitoring of both recreation pressures and wildlife dynamics (Fennell et al., 2022).

Wildlife responses to non‐consumptive recreation (e.g. hiking, motorized or non‐motorized recreation and horseback riding) vary widely (Larson et al., 2016) and may include behavioural avoidance of trails (Naidoo & Burton, 2020), use of trails primarily at night (Coppes et al., 2017), increased use of trails (Oriol‐Cotterill et al., 2015) or spatio‐temporal trail avoidance (Salvatori et al., 2023). The Human Shield Hypothesis (HSH) was proposed as a general mechanism to explain spatial responses by wildlife to human presence in wilderness areas, positing that mammalian carnivores avoid humans due to increased perceived mortality risk, which allows ungulates to use the predator‐free space near humans (Berger, 2007; Butler et al., 2021; Gámez & Harris, 2021; Muhly et al., 2011). Though observed at multiple study areas, it is not clear if prey and carnivores exhibit responses consistent with the HSH across varying levels of recreation and different ecological contexts. Predator density, forage availability, perceived risk and many other factors vary across space, so examining evidence for HSH at macroecological scales—spanning multiple landscapes and contexts—is important to test its generality (McGill, 2019).

Where wildlife and people share space, carnivores and prey may shift their temporal activity to avoid humans (Carter et al., 2012; Gaynor et al., 2018). Prey that select protective ‘shield’ areas might use recreation trails at night time, avoiding direct human interaction (Belotti et al., 2012; Gaynor et al., 2021). For example, red deer (*Cervus elaphus*) in a timber‐managed forest in Germany mainly used recreation trails at night when human activity was lower (Coppes et al., 2017). Similarly, in Colorado, USA, several species (mule deer, *Odocoileus hemionus*; black bear, *Ursus americanus*; coyote, *Canis latrans*) were more nocturnal on trails with more recreation activity (Lewis et al., 2021). Whether prey species respond to recreation through changes in diel overlap across landscapes and biomes is not well understood and it is not well known if land‐use disturbances (e.g. resource extraction) have an interactive effect on wildlife responses to recreation activity (Marion et al., 2021; Nickel et al., 2020; Sévêque et al., 2020).

Recreation is rarely the sole source of human disturbance in and around PAs that could affect wildlife. Wildlife responses to recreation could be influenced by habitat and land‐use context, such as the extent of other landscape disturbances like logging, fire, oil and gas extraction, and roads. All are widespread in western Canadian forests, contributing to the fragmentation and loss of natural wildlife habitat, potentially reducing large mammal abundances (Pickell et al., 2015; Shackelford et al., 2018; Venier et al., 2014). Vehicle collisions threaten wildlife and roads and are associated with habitat fragmentation while also increasing human access and recreation (Barrientos & Virgós, 2006; Coffin, 2007; Heagney et al., 2018). Increased accessibility to PAs near urban areas may be associated with large carnivore declines (Prugh et al., 2009) and human–wildlife conflict (Ditchkoff et al., 2006). However, the extent of the interaction between recreation and landscape disturbance has not been well tested (Nickel et al., 2020; Suraci et al., 2021), which makes it difficult to design conservation policies for recreation in wildlife areas.

Managing recreation to conserve biodiversity involves decision‐making over multiple spatial scales, land uses, and jurisdictions. Characterization of wildlife responses to human stressors may, in turn, vary with the spatial scale of analysis (Fisher et al., 2011; Toews et al., 2017), and it is often difficult to accurately inform broad‐scale management using results from single or small‐scale studies (Burton et al., 2014; Hobbs, 2003). More robust conclusions on the effects of recreation and other disturbances on wildlife can be obtained by synthesizing data across multiple study areas that span different contexts of habitat, land use, and human activity (Sensu Rich et al., 2017; Suraci et al., 2021). Large‐scale applications of new sampling technologies, such as camera trapping (Burton et al., 2015), can help overcome practical constraints that have limited the scope of previous studies, and ultimately improve our ability to test the generality of hypotheses about wildlife responses to growing human pressures (Chen et al., 2022).

We quantified wildlife responses to recreation across a gradient of land‐use disturbance (i.e. logging, roads) at two spatial scales and tested predictions of the HSH by synthesizing data from 10 camera trap arrays in western Canada. This region harbours one of the last intact large mammal assemblages in North America (Laliberte & Ripple, 2004; Shackelford et al., 2018; Westwood et al., 2019), providing an ideal system to investigate responses across a suite of large predators and prey. We expected responses to non‐consumptive recreation (hereafter, referred to as recreation) would be more negative and pronounced in habitats with greater disturbance from forest harvest and roads (Muhly et al., 2011). While consumptive recreation (hunting) does occur throughout our overall study area, we considered the effects to be relatively minor during our spring and summer sampling period, as hunting mainly takes place during the fall and winter months (Table S4). For carnivores, we predicted that species would minimize the perceived risk of mortality associated with human interaction (Gaynor et al., 2018; Oriol‐Cotterill et al., 2015) by avoiding areas where recreation and land‐use disturbances are prevalent as in Heinemeyer et al. (2019) and Reilly et al. (2017). We predicted that ungulate prey species would select areas with more human use to ‘shield’ themselves from predators (Berger, 2007; Kays et al., 2017; Muhly et al., 2011). We also tested the extent to which temporal responses to recreation varied with disturbance. We used mule deer—a commonly detected species across study areas—to test our prediction that even where ungulates show spatial overlap with recreation (as predicted by the HSH), they seek to minimize the probability of direct contact with humans and thus reduce diel activity overlap with recreation, particularly in the presence of more human habitat disturbance. Logging and roads can affect both predator and prey habitat use (Bowman et al., 2010; Colton et al., 2021; Prokopenko et al., 2017; Rogala et al., 2011), so we expected that activity overlap between people and mule deer would be lower where the risk of mortality is higher due to more vehicle traffic or greater visibility in the open habitats of logged areas (Lima & Dill, 1990; Rost & Bailey, 1979).

## 2 METHODS

### 2.1 Study area

**FIGURE 1:**
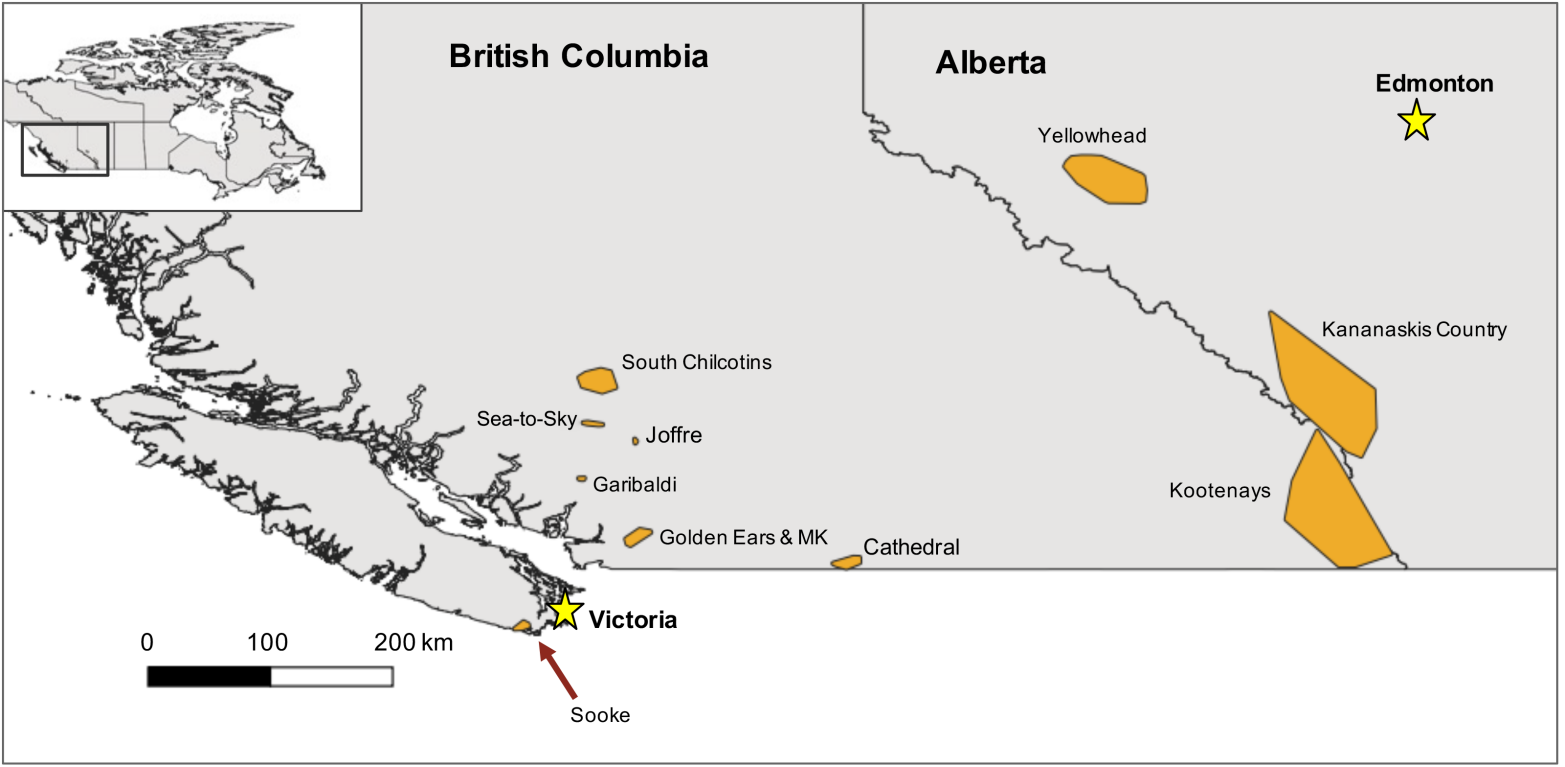
Geographical locations of camera arrays in British Columbia and Alberta included in our study: Sooke Capital Regional District (Klees van Bommel, 2022), Golden Ears Provincial Park and Malcolm Knapp Research Forest (Procko et al., 2022), Joffre Lakes Provincial Park, Garibaldi Provincial Park, Sea‐to‐Sky Mammal Monitoring Project (Dawe et al., Unpublished data), South Chilcotin Mountains (Naidoo & Burton, 2020), Cathedral Provincial Park (Fennell et al., 2022) Kootenay Remote Camera Wildlife Monitoring project (Chow, 2019), Kananaskis Country (Heim et al., 2019) and Yellowhead region (Ladle et al., 2018). Polygons represent minimum convex polygons around all camera stations in each study (buffered by 2 km). Also shown are capital cities for each province (Victoria, BC and Edmonton, AB).

Data were contributed by members of the WildCAM initiative (www.wildcams.ca), a western Canada‐based camera trap network established to facilitate coordinated regional sampling and analysis. Data came from 10 arrays, representing 446 camera trap sampling locations in British Columbia (BC) and Alberta (AB) (Figure 1). The study area spanned various ecoregions with similar wildlife communities (Demarchi, 2011) including Pacific Coast Mountain Ranges, Fraser River Valley, temperate conifer rainforest, Okanagan Range and eastern continental range (Government of Canada, 2013). Cameras were deployed in and around PAs within multiuse landscapes subject to varying levels of human disturbance including logging or roads in surrounding habitat. Sampling effort occurred between 2011 and 2021 with variable effort among projects (Table 1). Sampling designs between individual projects were variable as not all were initiated with the goal of estimating recreation. To reduce between‐array variation in survey designs and maintain our focus on the impacts of recreation, we included only camera stations that were set on linear features potentially used by humans (e.g. trails, roads) and excluded off‐trail deployments from analyses.

**TABLE 1:** Sampling details for on‐trail camera arrays included in this synthesis study.

Camera array	Camera stations	Total camera days	Mean days camera days per station	Min cam. distance (km)	Prop. baited cameras	Sampling design	Total MCP (km^2^)
Cathedral Prov. Park	17	3739	351	0.55	0	Stratified by human use	143.3
Golden Ears Provincial Park & Malcom Knapp	33	8678	195	1.06	0	Stratified by human use	165.9
Sea‐to‐Sky	10	1616	162	0.74	0	Stratified random	16.7
Joffre Lake Prov. Park	8	1791	224	0.48	0	Stratified by human use	2.7
Garibaldi Prov. Park	9	1753	195	0.37	0	Stratified by human use	4.7
Kootenays	18	2843	158	6.11	0	Systematic grid	6567.5
Sooke	25	3927	157	0.29	0	Stratified, Systematic grid	47.8
South Chilcotin Mountains Prov. Park	61	15,037	247	1.50	0	Systematic grid	511.1
Yellowhead region	227	30,621	135	0.89	0	Stratified random	2095.3
Kananaskis	38	9292	245	1.64	1	Systematic grid	6285.4

*Note*: Array names refer to general areas for distinct projects where data were collected. Data collection periods occurred from 2011 up to 2021. Sampling area describes the area of a minimum convex polygon encompassing all camera stations deployed on linear features potentially used by people (trails or roads). Sampling effort refers to days sampled within spring and summer months (April–September).

### 2.2 Study species

The HSH was developed with large‐bodied mammal species in mind (e.g. Berger, 2007; Muhly et al., 2011), and study designs for individual projects in our overall dataset were deployed with medium‐ to large‐bodied species as their focal taxa. We similarly focussed on large (>20 kg) mammals including ungulate prey (mule deer, *Odocoileus hemionus*; moose, *Alces alces*; and elk, *Cervus canadensis*) and large predators (wolf, *Canis lupus*; grizzly bear, *Ursus arctos*; black bear, *Ursus americanus*; and cougar, *Puma concolor*). Focal species were selected based on minimum thresholds of independent detections to allow multi‐area analysis, specifically ≥500 in total across ≥5 arrays. Detections of the same species at the same camera trap station were deemed independent if photographs were ≥30 min apart (Gerber et al., 2010; O'Brien et al., 2003).

### 2.3 Spatial responses to recreation and land‐use disturbance

To assess wildlife spatial responses to recreation and land‐use disturbance, we used Bayesian generalized linear mixed effect models (GLMMs) with a negative binomial distribution to account for overdispersion in the counts of detections (Equations 1 and 2). A Bayesian framework allowed us to model the relationship between wildlife detections and the magnitude of recreation at both local (individual camera trap stations) and landscape scales (all cameras in a given array), while assessing local‐scale impacts of land‐use disturbances (roads, logging), similar to approaches used by Rich et al. (2017) and Miller and Grant (2015). Our response variable was the number of independent detections per camera station for each focal ungulate and carnivore species, which we considered to be a measure of the frequency of site use over the sampling period (Beirne et al., 2021). For each focal species, we used one hierarchical model to assess species site use as a function of recreation, land use and environment.

**FIGURE 2:**
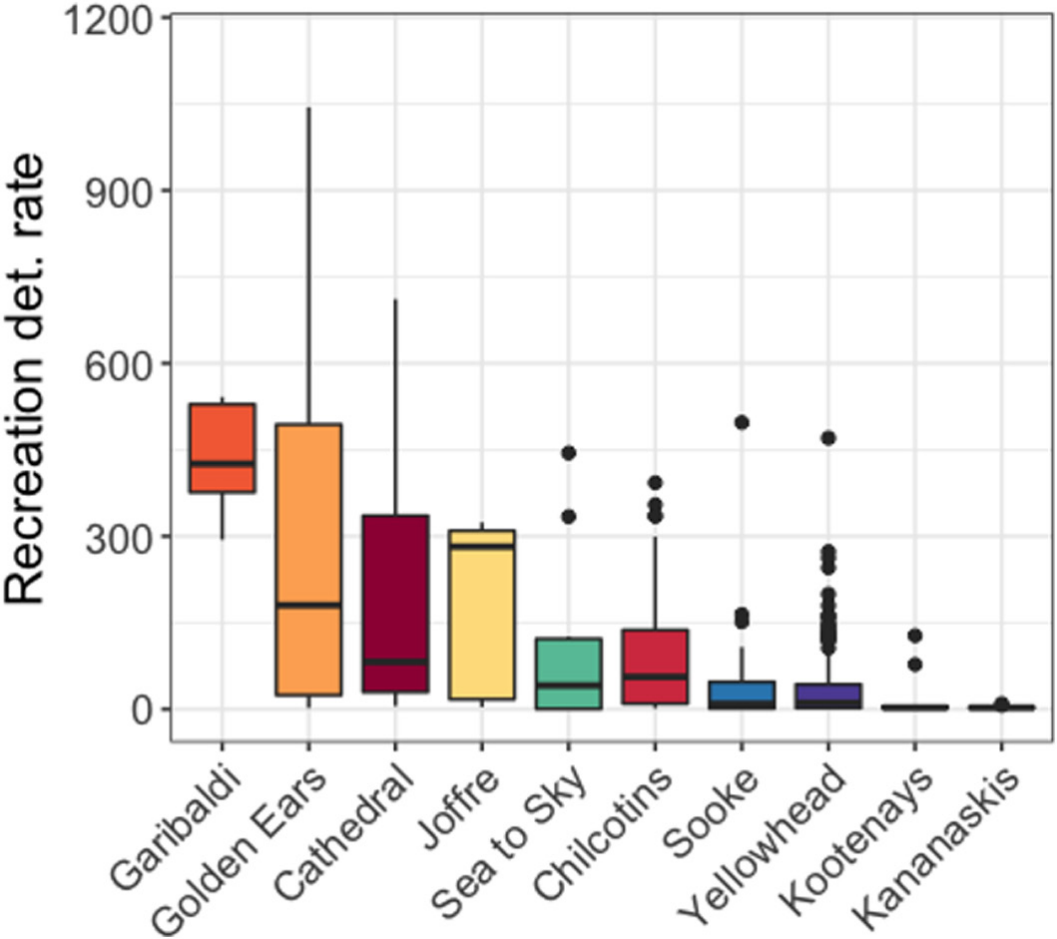
Boxplots illustrating range (median, interquartile range and outliers) of recreation detection rates (per 100 days) across camera trap stations on linear features within arrays in spring and summer months (April–September). Recreation detections include people on foot, motorized vehicles, cyclists, domestic dogs and horse riders. Detection rate is the number of independent detections of recreation, based on a 30‐min independence interval.

Cameras were active throughout the year, but recreation type and intensity can vary across seasons in western Canada. Visitation to PAs is typically higher in the spring and summer months relative to winter when snow makes some trails or parks less accessible. To account for the influence of seasonality, we focussed our spatial analyses on modelling wildlife detections in the spring and summer months (April–September), because there was greater variation in recreation activity during this period (Figure 2).

We defined recreation as non‐consumptive outdoor recreation activities, specifically measured by camera trap images of people on foot (with or without domestic dogs), motorized or non‐motorized vehicles or horseback riders. We acknowledge that consumptive recreation (hunting) can affect wildlife behaviour (e.g. Kays et al., 2017). Hunting of focal species occurs to varying degrees across the landscapes included in our study. In some areas, (Joffre, Garibaldi) hunting is totally prohibited, while in the others, seasonal hunting of some species is allowed (e.g. Sooke, Kananaskis Country, Table S4). Some of the humans detected by camera traps may have been hunters, but given that individual projects did not consistently distinguish between hunters and non‐hunters, we grouped human detections along with other types of recreation‐related activity at each site. Also, across camera arrays included in our study, hunting is largely seasonal and occurs outside of the spring–summer sample period. Thus, we also assumed most humans detected by our camera traps were not hunting. Recreation was modelled as a pooled detection rate (independent detections per 100 days) over the entire sampling period for each camera station.

To explore the effect of landscape disturbance context, we tested the influence of logging and road density on wildlife site use (Table 2, Figure S1). To improve model convergence, logging and road density were modelled as binary covariates (*high* and *low* levels of each, based on the extent of logged forest and of road density in a 500 m buffer around camera stations). *High* (≥10% logged area) and *low* (<10%) levels of logging were identified based on breaks in data. Similarly, *high* road density was classified as ≥1 km/km^2^ and *low* was determined as <1 km/km^2^ (Table 2). As human disturbances are unlikely to occur independently of one another, and multiple stressors can have cumulative impacts on wildlife (Burton et al., 2014), we also tested recreation × logging and recreation × road density interactions. We controlled for small‐scale differences in environmental context that may have affected wildlife detections at camera stations by including percent forest cover and normalized difference vegetation index (NDVI, from MODIS satellite, Table 2). We determined an NDVI value within a 250 m buffer around each camera station within seasons for each array using the R package *MODISTools* (Tuck et al., 2014). From each 16‐day NDVI value at camera stations, a total NDVI was determined for the overall time period when cameras were active during spring and summer months in a given array. We used the number of total days each camera station was active per season as a fixed effect to control for variation in sampling effort. Continuous covariates (i.e. detection rates, camera days and NDVI) were scaled to have a mean of 0 and standard deviation of 1. Correlations between covariates were tested with the *Hmisc* package in R (Harrell Jr., 2021). Percent forest cover was positively correlated with NDVI, so the former was excluded from analyses (*R* = .6, *p* < .05).

**TABLE 2:** Recreation, land use and habitat covariates used to model variation in site use of focal mammal species across 10 camera trap arrays in western Canada. All covariates were modelled at the camera trap station‐scale, except recreation, which was also modelled at the landscape scale.

Category	Covariate	Description	Source	
Recreation	Total recreation detection rate	Includes all types of recreation activity pooled (people on foot with or without domestic dogs, motorized recreation, cyclists, equestrian activity) per 100 days.	Camera trap data	
Land use	Logging	Total percent of area logged in the 50 years before sampling within a 500‐m radius of the station. Logging was modelled as binary covariate: *low* or *high* degree of logged area. Categories were determined through visual inspection of histogram of logging across camera stations. *High* logging is values >10% logged area; *Low* logging is values <10% logged area.	Harvested areas of BC^a^ Human Footprint data of AB^b^	
	Road	Road density (km/km^2^) in a 500‐m buffer around each camera station. For analyses, densities were modelled as binary covariate: *low* or *high* road density. Categories were determined through visual inspection of histogram of densities across camera stations. *High* road density includes values >1 km/km^2^; *Low* road density includes <1 km/km^2^	Digital Road Atlas^c^ National Road Network^d^ OpenStreetMap (OSM)^e^	
Habitat	NDVI	Normalized difference vegetation index (250‐m productivity), measured over the full period a camera station was active within seasons.	MODIS Satellite Product^f^	
	Percent forest cover	% of forested habitat within a 500‐m radius of the camera.	Land Cover of Canada^g^	
Control Variables	Camera days	Number of days all cameras were active within each season.	Camera trap data	

^a^ https://catalogue.data.gov.bc.ca/dataset/harvested‐areas‐of‐bc‐consolidated‐cutblocks.

^b^ https://www.abmi.ca/home/data‐analytics/da‐top/da‐product‐overview/Human‐Footprint‐Products/HF‐inventory.html.

^c^ https://www2.gov.bc.ca/gov/content/data/geographic‐data‐services/topographic‐data/roads.

^d^ https://open.canada.ca/data/en/dataset/3d282116‐e556‐400c‐9306‐ca1a3cada77f.

^e^ https://www.openstreetmap.org/.

^f^ MODIS Satellite Product (Tuck et al., 2014).

^g^ https://open.canada.ca/data/en/dataset/7f245e4d‐76c2‐4caa‐951a‐45d1d2051333.

GLMMs were structured to test the influence of local‐ and landscape‐scale covariates and tease apart scale‐dependent effects of recreation. This approach allows us to account for non‐independence of camera trap stations within arrays by including an index that links camera stations to the corresponding camera arrays (i.e., landscapes) in which they are nested.

For each species, the overall detection rate at camera station *i* was modelled as:

(1) $$\begin{array}{cc} \mathrm{Log}\;\left(\lambda_{i}\right)= & \;\beta_{1}*\;{\text{recreation}}_{i}+\beta_{2}*\;{\text{logging}}_{i}+\beta_{3}*\;{\text{road}}_{i}+\beta_{4}*\;{\text{camera}\;\text{days}}_{i}\\ & \;+\beta_{5}*\;\text{NDVI}\_{\text{season}}_{i}+\beta_{6}*\;\text{recreation}\times {\text{logging}}_{i}\\ & \;+\beta_{7}*\;\text{recreation}\times {\text{road}}_{i}+\varepsilon \;{\text{array}}_{j} \end{array}$$

The random effect for camera array *j* was, in turn, modelled as a function of recreation at the landscape scale as:

(2) $$ƒ\;\left(Ɛ{\text{array}}_{j}\right)={\beta_{8}}^{*}\;{\text{recreation}}_{j},$$

where *λ*_*i*_ represents the summed count of independent detections for each focal species in the spring and summer months, *β* represents the beta coefficient for each predictor variable and *Ɛ* denotes the random effect for camera array.

Models (one per species; seven in total) were run in R version 4.0.5 (R Core Team, 2021) with the package *jagsUI* (Kellner & Meredith, 2019) for 100,000 iterations. Each analysis saved posterior iterations from three chains with a prespecified thinning rate of 5 after discarding an initial burn‐in of 5000 iterations. Convergence was evaluated through visual inspection of trace plots and confirming R‐hat ≤1.1 (Brooks & Gelman, 1998). We used non‐informative priors with a uniform distribution for covariates and interpreted 95% credible intervals (CIs) that did not overlap with zero as providing strong evidence of the importance of that covariate in conditioning detection rates, while 80% CIs were interpreted as providing relatively weaker evidence (Kéry & Royle, 2020).

### 2.4 Temporal responses to recreation and land use

Analyses of temporal interactions between species require a large sample of detections at camera stations where those species co‐occur (Frey et al., 2017). Detections of elk and moose were insufficient at stations where they co‐occurred with recreation or with carnivores. Mule deer was the most frequently detected ungulate within arrays and was chosen as our focal ungulate prey species to test for reduced activity overlap with recreation and carnivores in disturbed habitats.

To characterize diel activity, the time stamp of each independent detection event for mule deer was converted to solar time to account for differences in day length among arrays (Frey et al., 2020; Nouvellet et al., 2012). Solar times were then converted to radians (2π radians = 24). We determined the coefficient of activity overlap between mule deer and (i) recreation activity and (ii) carnivores, at each camera trap station using the *activity* R package (Rowcliffe, 2016). The number of overall detections for mule deer and for each group (carnivores, recreators) used to calculate activity overlap for each comparison was ≥75, so we used the overlap ∆_4_ to estimate which ranges between 0 (no overlap in activity) and 1 (100% overlap) (Meredith & Ridout, 2016; Ridout & Linkie, 2009).

Overlap coefficient calculations can be sensitive to sample size (Lashley et al., 2018) and activity calculations can be unreliable where detections are rare. We thus used data from all months (i.e. data from all seasons pooled) in our temporal analysis while only including data from individual stations with ≥25 detections of both species in each pair (i.e. mule deer and carnivores, mule deer and recreationists), balancing the need to maintain a sufficient number of locations while minimizing potential error associated with stations where both or one species rarely occurred or were absent (Frey et al., 2020).

Species activity patterns may be influenced by anthropogenic habitat disturbances (Frey et al., 2020; Wang et al., 2015). Using an approach similar to Frey et al. (2020) and Wang et al. (2015), we tested the influence of logging and road density on activity overlap (see Section 2.3 and Table 2 for details on covariates). To test the influence of logging and road density on activity overlap, we ran Bayesian linear mixed effect models (LMMs) in *jagsUI* (Kellner & Meredith, 2019) with camera station overlap coefficients as the response variable. Overlap between mule deer and recreation was modelled as a function of road density, logging, and carnivore detection rate (Equation 3). Mule deer–carnivore overlap was modelled as a function of road density, logging and recreation detection rate (Equation 4). Carnivore detection rate includes pooled detections of multiple species that are known to predate on mule deer adults or fawns to varying degrees (black bear, grizzly bear, cougar, wolf, coyote [*Canis latrans*]). The number of detections for each was also included as a covariate in the models (mule deer *n* = number of mule deer detections; recreation *n* = number of recreation detections; carnivore *n* = carnivore detections). This resulted in a total of 64 and 79 camera locations for the mule deer–recreation overlap and mule deer–carnivore overlap analyses, respectively. Camera array was modelled as a random intercept in LMMs. As in our spatial analyses, our approach allowed us to include an index that links stations to the corresponding arrays in which they are nested. We ran each model with three chains of 100,000 iterations each, a thin rate of 3, burn‐in period of 5000 and random initial start values.

Overlap coefficients for mule deer‐recreation at each camera station *i* were modelled with LMMs as:

(3) $$\begin{array}{cc} {\text{Overlap coefficient}}_{i}= & \;{\beta_{2}}^{*}\;{\text{logging}}_{i}+{\beta_{3}}^{*}\;{\text{road}}_{i}\\ & \;+{\beta_{8}}^{*}\;\text{carnivore}\;\det\;{\text{rate}}_{i}+{\beta_{9}}^{*}\;\text{mule deer}\_n_{i}\\ & \;+{\beta_{10}}^{*}\;\text{recreation}\_n_{i}+\varepsilon \;{\text{array}}_{j} \end{array}$$

For mule deer–carnivore, overlap at camera station *i* was modelled as

(4) $$\begin{array}{cc} {\text{Overlap coefficient}}_{i}= & \;{\beta_{1}}^{*}\;{\text{recreation}}_{i}+{\beta_{2}}^{*}\;{\text{logging}}_{i}+{\beta_{3}}^{*}\;{\text{road}}_{i}\\ & \;+{\beta_{9}}^{*}\;\text{mule deer}\_n_{i}+{\beta_{12}}^{*}\;\text{carnivore}\_n_{i}\\ & \;+\varepsilon \;{\text{array}}_{j}, \end{array}$$

where *β* represents the beta coefficient for each predictor variable and *Ɛ* denotes the random effect for camera array.

## 3 RESULTS

### 3.1 Wildlife and human activity across landscapes

Camera trap sampling revealed wide variation in the intensity of recreational activity (i.e. human detections) across camera arrays (Figure 2). Sampled areas within Golden Ears and Garibaldi Provincial parks in BC had the highest overall and most variable recreation detection rates, while the more eastern landscapes of Kootenays in BC and Kananaskis in AB had the least amount of recreation detections (Figure 2).

Overall, detection rates for focal wildlife species were highest in South Chilcotin Mountains Provincial Park (BC) and in the Yellowhead and Kootenays landscapes (AB), with some variation across species (detection rates for individual species are shown in Figures S2 and S3). Black bears were the most frequently detected carnivore overall, whereas mule deer were the most frequently detected ungulate (Figures S2 and S3).

### 3.2 Spatial responses to recreation and landscape disturbance

**FIGURE 3:**
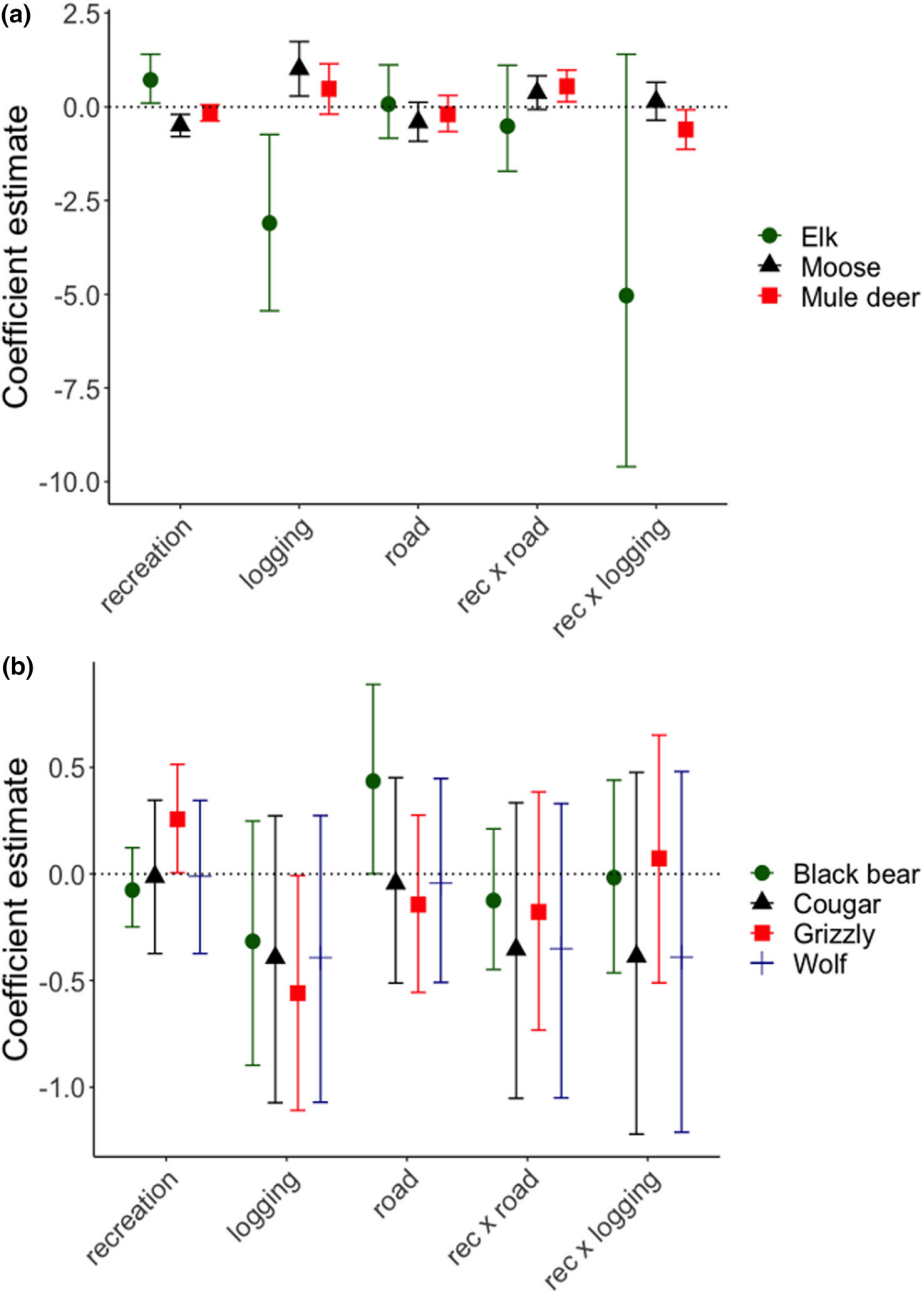
Recreation and land‐use disturbance‐related coefficient estimates and 95% credible intervals (CI) from hierarchical models testing the effects of recreation, road density and logging on (a) ungulate and (b) carnivore habitat use. Covariates shown here were modelled at the local (i.e. camera station level) scale. Details about each covariate are included in Table 2. Reference levels for logging and road density covariates are *high* for each. Detection rates are per 100 days. Coefficient estimates for all other covariates tested are shown in Tables S1 and S2.

Species did not respond strongly to variation in recreation at the landscape scale, but wildlife use at the local (camera) scale strongly varied with disturbance and recreation (Tables S1 and S2, Figure 3). Among ungulates, only elk site use was positively associated with recreation (*β*_1_, mean posterior estimate = 0.72, 95% CI: 0.10–1.41), while for moose, this relationship was negative (*β*_1_ = −0.49, 95% CI: −0.79 to −0.21). Mule deer site use was also negatively affected by local recreation (*β*_1_ = −0.17, 80% CI: −0.31 to −0.03), but at the landscape scale, recreation had a positive effect on this species (*β*_8_ = 0.59, 80% CI: 0.19–0.98). For carnivores, landscape‐scale recreation only affected black bears, though this relationship was positive (*β*_8_ = 0.28, 80% CI: 0.10–0.45). Grizzly bears responded more strongly to local recreation, and this relationship was also positive (*β*_1_ = 0.26, 95% CI: 0.01–0.51).

Species responded to other local‐scale land use disturbances. Logging at camera stations affected moose and elk site use, though in opposite ways. Moose site use was greater at highly logged sites (*β*_3_ = 1.10, 95% CI; 0.29–1.74), whereas for elk, site use was greater at low logging sites (*β*_3_ = −3.10, 95% CI: −5.44 to −0.74). Logging and road density also affected local site use by bears. For black bears, site use was greater at *high* road density sites (*β*_3_ = 0.44, 95% CI: 0.001–0.89). For grizzly bears, site use was reduced in areas with *high* logging (grizzly: *β*_2_ = −0.56, 95% CI: −1.11 to −0.001).

We detected strong interactions between recreation and land use disturbances. Even though mule deer site use did not strongly vary with road density, it was affected by a recreation × road density interaction. Mule deer site use was reduced in areas with *high* logging and recreation (*β*_6_ = −0.60, 95% CI: −1.14 to −0.08) while site use increased at *high* road density sites with recreation (*β*_7_ = 0.55, 95% CI: 0.14–0.98).

Our control variables affected species presence to varying degrees. NDVI had a strong negative association with site use for elk (*β*_5_ = −0.68, 95% CI: −1.01 to −0.38), while we found the opposite trend of greater site use in areas with higher overall NDVI for moose (*β*_5_ = 0.31, 95% CI: 0.15–0.47), black bear (*β*_5_ = 0.69, 95% CI: 0.55–0.84) and cougar (*β*_5_ = 0.25, 95% CI: 0.09–0.41) (Tables S2 and S3). For mule deer, wolves and grizzly bears, we detected no effect of NDVI on site use. Sampling effort (camera days) had a strong and positive effect on site use for all species, except for cougars (Tables S2 and S3).

### 3.3 Temporal responses to recreation

**FIGURE 4:**
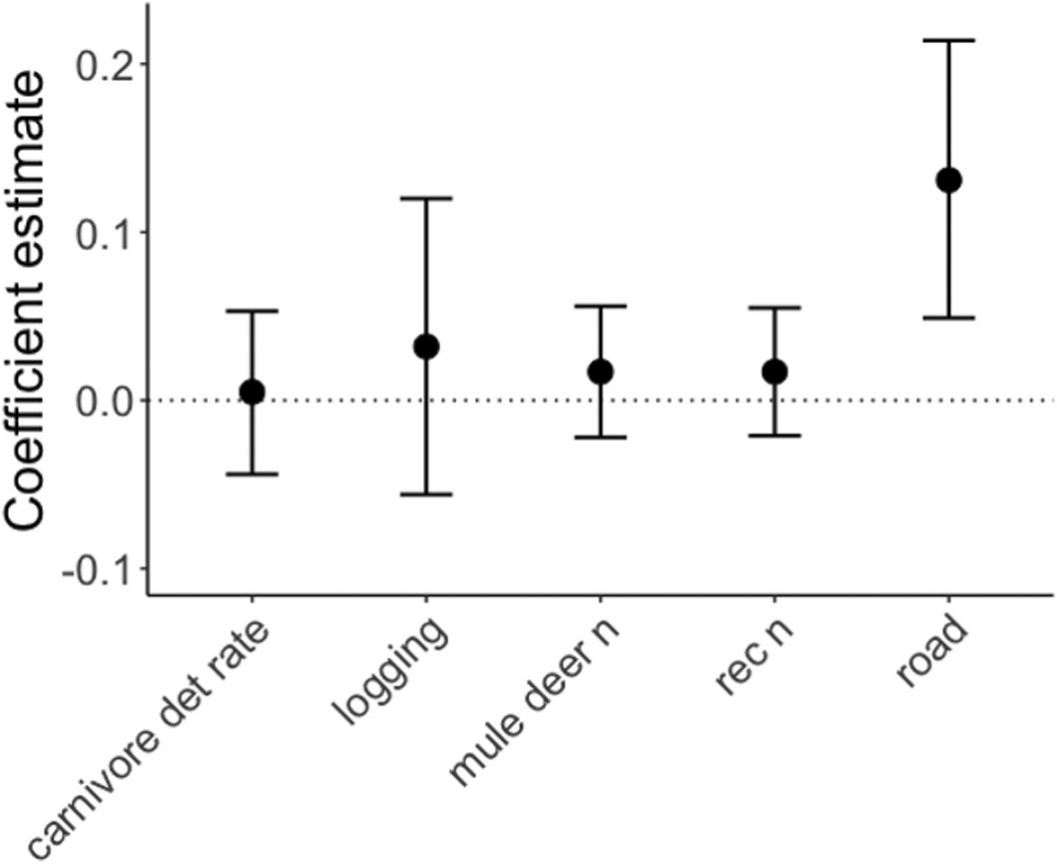
Estimates from linear mixed effect models testing the overall influence of recreation detection rate, land‐use disturbance (road density, logging) and carnivore detection rate (per 100 days) on the coefficient of activity overlap for mule deer and recreation activity. Carnivore detection rate includes pooled independent detections of cougar, black bear, grizzly bear, wolf and coyote. Reference levels for logging and road density covariates are *high* for each. Model estimates and 95% CI values are included in Table S3.

The coefficient of activity overlap (Δ_4_) throughout the whole year between mule deer and recreation activity was 0.60, indicating moderate overlap in timing of activity (Figure S4, seasonal overlap curves shown in Figure S5). Overlap between mule deer and recreation activity was higher at camera stations in areas with *high* road density (*β*_3_ = 0.13, 95% CI: 0.05–0.21), but overlap was unaffected by logging or carnivore detection rate (Table S3). With respect to overlap between mule deer and carnivores, the overlap coefficient was unaffected by recreation or by logging, though there was some evidence that overlap between mule deer and carnivores was also greater in areas with *high* road density (*β*_3_ = 0.05, 80% CI 0.02 to 0.09; Table S3; Figure 4).

## 4 DISCUSSION

Recreation can have important impacts on wildlife occurrence and co‐occurrence patterns at camera sites within landscapes (Naidoo & Burton, 2020). As past studies of the HSH have been constrained to single landscapes (e.g. Muhly et al., 2011), we have not known whether this behavioural phenomenon is generalizable across different landscapes and environmental contexts. In our hierarchical approach with camera traps distributed across 10 landscapes in western Canada, we found limited support for the HSH at macroecological scales, suggesting it is not a general phenomenon, but a local one.

Under the HSH, carnivores are expected to avoid recreation, given the increased risk of anthropogenic mortality (Berger, 2007), but contrary to our predictions, grizzly bear site use was positively associated with recreation. Recreation might affect some individuals more than others, and the effects may vary by landscape context. For example, mothers and cubs increased their movement rates but did not avoid recreation trails in AB (Ladle et al., 2018). They were even less likely to avoid trails with non‐motorized recreation, which were often in more rugged terrain. This may have forced bears to use the trails, more so than in less rugged areas with motorized recreation (Finnegan et al., 2018; Ladle et al., 2018). Grizzly bears and people may be drawn to similar habitats, such as high‐productivity areas at valley bottoms, and high‐quality food near linear features, leading to increased spatial overlap and potentially, to more human–wildlife conflict (Elmeligi et al., 2021; Finnegan et al., 2018; Gibeau et al., 2001; Ladle et al., 2018). Thus, while spatial avoidance of people by bears has been documented (Fortin et al., 2016), a lack of spatial avoidance does not necessarily indicate sustainable coexistence, and a more nuanced analysis of individual fitness and population demographics is warranted. Suraci et al. (2021) also found that site use for other carnivores increased with human presence (e.g. recreation), though this may reflect the increasing prevalence of recreation in wildlife habitat, rather than selection for areas with recreation. Our results are contrary to those from one of our single landscapes, where Naidoo and Burton (2020) found fewer grizzly bear detections where mountain biking was more prevalent in South Chilcotin Mountains Provincial Park (BC). This signals different patterns manifesting at landscape versus regional spatial scales. Pattern differences may also arise across temporal scales: their analyses were performed at a finer (i.e. weekly) temporal scale suggesting that grizzlies might share space over long time periods (seasons), while partitioning weekly time with humans (Naidoo & Burton, 2020). Similarly, Fortin et al. (2016) found that brown bears were spatially and temporally displaced from recreation which was associated with reduced feeding and increased energetic costs.

Human activity is expected to alter habitat use by ungulates under the HSH, with ungulates selecting areas with increased recreation because they are ‘shielded’ from predators or from hunting by non‐compensatory human activity (Berger, 2007). The lack of the predicted, consistently positive responses of ungulates to recreation may be associated with a lack of predicted negative responses by carnivores. At the landscape scale, only mule deer site use was positively affected by recreation, though this was weaker than local scale responses, highlighting the need for more large‐scale studies measuring ungulate response to recreation across landscapes. Elsewhere, large‐scale responses by ungulates were variable, with stronger positive responses detected. Positive responses to recreation by ungulates were also detected in North America (Suraci et al., 2021) but not in Germany (Coppes et al., 2017). At the local scale, ungulates in our study (except elk) did not select areas with increased human activity, contrary to predictions and to findings from single landscapes in Europe (Westekemper et al., 2018) and elsewhere in North America (Baker & Leberg, 2018; Muhly et al., 2011; Rogala et al., 2011). Moose site use decreased with recreation as in Sytsma et al. (2022) and Neumann et al. (2011) and similarly, mule deer site use was negatively but weakly associated with local recreation.

Other anthropogenic disturbances seemed to have stronger effects on species than did recreation. Moose site use increased in more intensely logged habitat, suggesting that foraging opportunities in open habitat could outweigh the risks associated with increased visibility and predation in those areas (Boucher et al., 2009; Francis et al., 2021; Massé & Côté, 2009). For carnivores, responses to other forms of human disturbance varied among species, as was demonstrated in one of our single landscapes (Kananaskis, Heim et al., 2019). Some carnivores showed strong responses to landscape disturbance variables, though the direction of these relationships were variable. Black bear site use was positively associated with *high* road density. However, this too may be a function of ‘cascading’ risk perception as black bears segregate from grizzly bears (Ladle et al., 2018).

Despite the lack of strong responses towards recreation on its own, there were significant interactions between recreation and other disturbance variables tested. Mule deer site use was associated with the interacting effects of recreation with logging and with road density and may provide partial support for the HSH. If perceived predation risk is reduced in open areas (e.g. areas near roads) also subject to recreation, ungulates may benefit from using those areas (Tinoco Torres et al., 2011). Conversely, the interacting effects of recreation and logging were negatively related to mule deer site use. For mule deer in these landscapes, the risks associated with being in more open habitats created by logging could be greater than that of road density. Thus, the HSH may be less likely supported where logging has occurred, possibly because of impacts on vegetation or forest structure created by timber extraction that differ from those created by road creation. In summary, the behavioural responses in the HSH are variably expressed in space. Also, these responses depend on the contexts imparted by anthropogenic landscape disturbance and how it affects perceived risk, and true risk from carnivores.

Studies that test hypotheses like the HSH (Berger, 2007) and make predictions about how predators and prey shift their activity to minimize risk from human disturbance and predation could guide management efforts of predators and prey in PAs—but only if we understand the variability around when, and where, the HSH manifests. Assessing animal site use at macroecological spatial scales encompassing multiple landscapes with different contexts (McGill, 2019) can identify behaviours that facilitate or impede coexistence in human‐occupied systems. Increased daily overlap with non‐consumptive recreation could lead to direct interactions with humans, and potentially, human–wildlife conflict, while landscape disturbances might also affect activity patterns after extended exposure to human recreation (Taylor & Knight, 2003). Overlap between mule deer and recreation increased with high road density, and there was a similar (though weak) positive relationship for overlap between mule deer and carnivore activity. This suggests that deer may alter activity patterns in disturbed areas (Frey et al., 2020) and may perceive less risk from recreationists in areas with roads relative to undisturbed habitat (Lone et al., 2014). However, we caution against using this trend to design management where consumptive recreation is prevalent (e.g. hunting), which can negatively affect wildlife through displacement (Kays et al., 2017). Hunting seasons or areas may create a more predictable landscape of fear which can result in strong wildlife behavioural responses (Cromsigt et al., 2013), and wildlife may even show stronger reactions to hunting than towards predators (Ciuti et al., 2012). Further research into the effects of consumptive recreation relative to those of non‐consumptive recreation and their potential interactive effects is needed.

## 5 CONCLUSIONS

Outdoor recreation is increasingly recognized as a potentially negative influence on wildlife site use. Synthesizing data across multiple scales and gradients of interest is a powerful tool for testing the generality of wildlife responses to human disturbances (Chen et al., 2022; Khwaja et al., 2019; Rich et al., 2017), including recreation. Our results highlight the species‐ and context‐dependency of human–wildlife interactions in increasingly anthropogenic landscapes, which can affect inferences about general wildlife responses to stressors. Species might respond differently to recreation based on historical human pressures. Some species (e.g. grizzly bear, wolf) have already been locally extirpated or are rarely detected, which might contribute to increased prevalence of human‐tolerant species (species ‘filtering’). Others may exhibit density‐dependent behaviours influenced by contemporary human disturbance (Morehouse et al., 2016; O'Neil et al., 2020). Conservation effectiveness could be impacted if the effects of recreation are considered in isolation of co‐occurring disturbance.

Recreation can cause changes in animal habitat use, and growing recreation pressure is part of the cumulative impacts to wildlife in the Anthropocene. We emphasize the need for expanded macroecological‐scale research of both human and wildlife use of recreation areas, particularly with respect to different types and quantities of recreation (e.g. motorized and non‐motorized recreation, skiing, cycling, camping and hunting) (Kays et al., 2017; Naidoo & Burton, 2020; Parsons et al., 2022) and in different contexts of land‐use management. Conservation efforts are increasingly focussing on the expansion of PAs across the globe (Watson et al., 2014), so finding ways that human activities can be compatible with conservation should be a priority in order to facilitate human–wildlife coexistence. Given the ever‐increasing pressure on PAs, much more research into human– wildlife coexistence in space and time is needed.

## AUTHOR CONTRIBUTIONS

**Alys Granados:** Conceptualization (equal); data curation (equal); formal analysis (lead); methodology (lead); project administration (equal); writing – original draft (lead); writing – review and editing (lead). **Catherine Sun:** Conceptualization (supporting); formal analysis (supporting); methodology (supporting); writing – review and editing (equal). **Jason T. Fisher:** Conceptualization (supporting); data curation (equal); funding acquisition (equal); project administration (equal); writing – review and editing (equal). **Andrew Ladle:** Data curation (equal); formal analysis (equal); investigation (equal); writing – review and editing (equal). **Kimberly Dawe:** Data curation (equal); investigation (equal); writing – review and editing (equal). **Christopher Beirne:** Writing – review and editing (equal). **Mark S. Boyce:** Data curation (equal); funding acquisition (equal); writing – review and editing (supporting). **Emily Chow:** Data curation (equal); investigation (equal); writing – review and editing (equal). **Mitchell Fennell:** Data curation (equal); investigation (equal); writing – review and editing (equal). **Nicole Heim:** Data curation (equal); investigation (equal). **Joanna Klees van Bommel:** Data curation (equal); investigation (equal); writing – review and editing (supporting). **Robin Naidoo:** Data curation (equal); funding acquisition (equal); investigation (equal); writing – review and editing (supporting). **Michael Procko:** Data curation (equal); investigation (equal); writing – review and editing (equal). **Frances E. C. Stewart:** Data curation (equal); funding acquisition (equal); investigation (equal); writing – review and editing (equal). **A. Cole Burton:** Conceptualization (equal); data curation (equal); formal analysis (supporting); funding acquisition (lead); methodology (equal); project administration (equal); supervision (lead); visualization (equal); writing – original draft (supporting); writing – review and editing (equal).

## CONFLICT OF INTEREST STATEMENT

None to declare.

## Supporting information


Data S1

## Data Availability

Data and R code are available online on https://figshare.com/authors/Alys_Granados/4108249.
