# Correction to Granados et al. 2023. Mammalian predator and prey responses to recreation and land use across multiple scales provide limited support for the human shield hypothesis, Ecology and Evolution, 13: 10464

**DOI:** 10.1002/ece3.10596

**Published:** 2023-10-05

The original article included incorrect author affiliations for some of the authors. Below is the correct list of affiliations for each.

Alys Granados, Department of Forest Resources Management, University of British Columbia, Vancouver, British Columbia, Canada.

Catherine Sun, Department of Forest Resources Management, University of British Columbia, Vancouver, British Columbia, Canada.

Jason T. Fisher, School of Environmental Studies, University of Victoria, Victoria, British Columbia, Canada.

Andrew Ladle, School of Environmental Studies, University of Victoria, Victoria, British Columbia, Canada.

Kimberly Dawe, Quest University Canada, Squamish, British Columbia, Canada.

Christopher Beirne, Department of Forest Resources Management, University of British Columbia, Vancouver, British Columbia, Canada.

Mark S. Boyce, Department of Biological Sciences, University of Alberta, Edmonton, Alberta, Canada.

Emily Chow, British Columbia Ministry of Forests, Lands, Natural Resource Operations and Rural Development, Cranbrook, British Columbia, Canada.

Mitchell Fennell, Department of Forest Resources Management, University of British Columbia, Vancouver, British Columbia, Canada.

Nicole Heim, Ktunaxa Nation Government, Cranbrook, British Columbia, Canada.

Joanna Klees van Bommel, Department of Forest Resources Management, University of British Columbia, Vancouver, British Columbia, Canada.

Robin Naidoo, World Wildlife Fund‐US, Washington DC, USA; Institute for Resources, Environment and Sustainability, University of British Columbia, Vancouver, British Columbia, Canada.

Michael Procko, Department of Forest Resources Management, University of British Columbia, Vancouver, British Columbia, Canada.

Frances E.C. Stewart, Department of Biology, Wilfrid Laurier University, Waterloo, Ontario, Canada.

A. Cole Burton, Department of Forest Resources Management and Biodiversity Research Centre, University of British Columbia, Vancouver, British Columbia, Canada.

We apologize for this error.

