# Supplementary material for: Mammalian predator and prey responses to recreation and land use across multiple scales provide limited support for the human shield hypothesis
Source: Ecol Evol. 2023 Sep 14;13(9):e10464. doi: 10.1002/ece3.10464 (PMC10500421; doi:10.1002/ece3.10464)
Supplement: Supplementary file 1 — Data S1 [file ECE3-13-e10464-s001.docx]

**SUPPLEMENTARY INFORMATION**

***Mammalian predator and prey responses to recreation and land use across multiple scales provide limited support for the human shield hypothesis***

Alys Granados

Catherine Sun

Jason T. Fisher

Andrew Ladle

Kimberley Dawe

Christopher Beirne

Mark S. Boyce

Emily Chow

Mitchell Fennell

Nicole Heim

Joanna Klees van Bommel

Robin Naidoo

Michael Procko

Frances E.C. Stewart

A. Cole Burton

**Supplementary Tables**

**Spatial analysis model estimate tables**

**Table S1**. Mean posterior estimates and 95% and 80% credible intervals (CIs) from general linear mixed effect models testing the effects of recreation, disturbance, habitat, and sampling effort on **ungulate** habitat use at camera trap stations deployed on linear features (trails or road) in spring and summer months (April to September). Reference levels for road density (road) and logging variables are *High* levels of each. The spatial scale (local, landscape) at which each covariate was model is also noted. Details about covariates are provided in Table 2.

| Species | Spatial Scale | Covariate | mean | sd | median | 80% BCI | 95% BCI | Rhat | n.eff |
| --- | --- | --- | --- | --- | --- | --- | --- | --- | --- |
| Mule deer | local | logging [high] | 0.478 | 0.343 | 0.482 | **0.036, 0.917** | -0.199, 1.144 | 1 | 19605 |
| Mule deer | local | days | 0.743 | 0.083 | 0.742 | **0.637, 0.848** | **0.580, 0.904** | 1 | 39245 |
| Mule deer | local | NDVI | 0.017 | 0.067 | 0.018 | -0.069, 0.103 | -0.115, 0.146 | 1 | 33611 |
| Mule deer | local | road [high] | -0.195 | 0.246 | -0.201 | -0.507, 0.122 | -0.663, 0.301 | 1 | 57000 |
| Mule deer | local | recreation | -0.168 | 0.111 | -0.171 | **-0.307, -0.026** | -0.376, 0.057 | 1 | 47481 |
| Mule deer | local | rec x road | 0.545 | 0.215 | 0.538 | **0.274, 0.822** | **0.136, 0.982** | 1 | 57000 |
| Mule deer | local | rec x logging | -0.604 | 0.270 | -0.601 | **-0.949, -0.261** | **-1.136, -0.079** | 1 | 57000 |
| Mule deer | landscape | mean rec. | 0.588 | 0.322 | 0.586 | **0.190, 0.983** | -0.049, 1.235 | 1 | 57000 |
| Elk | local | logging [high] | -3.103 | 1.221 | -3.117 | **-4.680, -1.510** | **-5.440, -0.740** | 1 | 25557 |
| Elk | local | days | 0.794 | 0.159 | 0.792 | **0.592, 0.997** | **0.489, 1.116** | 1 | 57000 |
| Elk | local | NDVI | -0.680 | 0.161 | -0.677 | **-0.889, -0.475** | **-1.008, -0.376** | 1 | 36724 |
| Elk | local | road [high] | 0.072 | 0.495 | 0.048 | -0.539, 0.715 | -0.835, 1.116 | 1 | 30500 |
| Elk | local | recreation | 0.718 | 0.333 | 0.706 | **0.301, 1.151** | **0.100, 1.405** | 1 | 57000 |
| Elk | local | rec x road | -0.515 | 0.601 | -0.509 | -1.280, 0.238 | -1.717, 1.106 | 1 | 57000 |
| Elk | local | rec x logging | -5.035 | 2.613 | -0.507 | **-8.610, 1.40**1 | -9.600, 1.405 | 1 | 35407 |
| Elk | landscape | mean rec. | -0.299 | 0.447 | -0.273 | -0.835, 0.203 | -1.252, 0.662 | 1 | 57000 |
| Moose | local | logging [high] | 1.013 | 0.371 | 1.014 | **0.536, 1.486** | **0.285, 1.741** | 1 | 33082 |
| Moose | local | days | 0.700 | 0.087 | 0.699 | **0.589, 0.811** | **0.531, 0.873** | 1 | 14913 |
| Moose | local | NDVI | 0.312 | 0.082 | 0.312 | **0.207, 0.417** | **0.150, 0.473** | 1 | 57000 |
| Moose | local | road [high] | -0.408 | 0.266 | -0.412 | -0.745, -0.067 | -0.917, 0.122 | 1 | 44107 |
| Moose | local | recreation | -0.494 | 0.149 | -0.493 | **-0.687, -0.304** | **-0.792, -0.205** | 1 | 57000 |
| Moose | local | rec x road | 0.371 | 0.227 | 0.370 | **0.085, 0.661** | **-0.070, 0.826** | 1 | 20308 |
| Moose | local | rec x logging | 0.142 | 0.256 | 0.140 | -0.192, 0.470 | -0.356, 0.653 | 1 | 57000 |
| Moose | landscape | mean rec. | 0.171 | 0.200 | 0.172 | -0.070, 0.410 | -0.228, 0.564 | 1 | 57000 |

**Table S2**. Mean posterior estimates and 95% and 80% credible intervals (CIs) from general linear mixed effect models testing the effects of recreation, disturbance, habitat, and sampling effort on **carnivore** habitat use during spring and summer months at camera trap stations deployed on linear features (trails or road). Reference levels for road density (road) and logging variables are *High* levels of each. The spatial scale (local, landscape) at which each covariate was model is also noted. Details about covariates are provided in Table 2.

| Species | Spatial Scale | Covariate | mean | sd | median | 80% CI | 95% CI | Rhat | n.eff |
| --- | --- | --- | --- | --- | --- | --- | --- | --- | --- |
| Black bear | local | logging [high] | -0.316 | 0.292 | -0.312 | -0.693, 0.056 | -0.898, 0.248 | 1 | 45945 |
| Black bear | local | days | 0.75 | 0.077 | 0.749 | **0.652, 0.848** | **0.601, 0.903** | 1 | 57000 |
| Black bear | local | NDVI | 0.69 | 0.074 | 0.689 | **0.596, 0.784** | **0.547, 0.835** | 1 | 57000 |
| Black bear | local | road [high] | 0.435 | 0.226 | 0.433 | **0.149, 0.726** | **0.001, 0.889** | 1 | 33457 |
| Black bear | local | recreation | -0.076 | 0.095 | -0.08 | -0.193, 0.049 | -0.249, 0.123 | 1 | 26402 |
| Black bear | local | rec x road | -0.125 | 0.168 | -0.127 | -0.338, 0.089 | -0.448, 0.211 | 1 | 57000 |
| Black bear | local | rec x logging | -0.017 | 0.231 | -0.019 | -0.310, 0.279 | -0.464, 0.440 | 1 | 57000 |
| Black bear | landscape | mean rec. | 0.275 | 0.142 | 0.275 | **0.100, 0.451** | -0.008, 0.555 | 1 | 57000 |
| Cougar | local | logging [high] | -0.393 | 0.343 | -0.39 | -0.832, 0.048 | -1.073, 0.273 | 1 | 57000 |
| Cougar | local | days | 0.675 | 0.083 | 0.675 | -0.932, 0.781 | 0.515, 0.840 | 1 | 54031 |
| Cougar | local | NDVI | 0.249 | 0.084 | 0.249 | **0.569, 0.355** | **0.085, 0.413** | 1 | 57000 |
| Cougar | local | road [high] | -0.044 | 0.245 | -0.047 | -0.354, 0.271 | -0.512, 0.451 | 1 | 32143 |
| Cougar | local | recreation | -0.013 | 0.184 | -0.013 | -0.245, 0.222 | -0.373, 0.346 | 1 | 37443 |
| Cougar | local | rec x road | -0.354 | 0.352 | -0.353 | -0.803, 0.095 | -1.053, 0.334 | 1 | 34341 |
| Cougar | local | rec x logging | -0.387 | 0.432 | -0.393 | -0.936, 0.166 | -1.221, 0.476 | 1 | 57000 |
| Cougar | landscape | mean rec. | -0.177 | -1.396 | -0.164 | -0.872, 0.503 | -0.164, 0.974 | 1 | 10537 |
| Wolf | local | logging [high] | -0.393 | 0.344 | -0.390 | -0.834, 0.043 | -1.071, 0.274 | 1 | 35445 |
| Wolf | local | days | 0.675 | 0.083 | 0.674 | **0.568, 0.781** | **0.514, 0.841** | 1 | 57000 |
| Wolf | local | NDVI | 0.248 | 0.084 | 0.249 | **0.141, 0.355** | **0.082, 0.411** | 1 | 22725 |
| Wolf | local | road [high] | -0.043 | 0.244 | -0.047 | -0.355, 0.277 | -0.509, 0.447 | 1 | 29008 |
| Wolf | local | recreation | -0.012 | 0.184 | -0.010 | -0.248, 0.220 | -0.373, 0.345 | 1 | 12372 |
| Wolf | local | rec x road | -0.351 | 0.351 | -0.349 | -0.799, 0.084 | -1.051, 0.330 | 1 | 20656 |
| Wolf | local | rec x logging | -0.391 | 0.431 | -0.398 | -0.930, 0.167 | -1.212, 0.480 | 1 | 57000 |
| Wolf | landscape | mean rec. | -0.174 | 0.584 | -0.146 | -0.867, 0.515 | -1.383, 1.007 | 1 | 57000 |
| Grizzly | local | logging [high] | -0.559 | 0.28 | -0.559 | **-0.918, -0.201** | **-1.109, -0.001** | 1 | 52838 |
| Grizzly | local | days | 0.76 | 0.071 | 0.76 | **0.669, 0.852** | **0.622, 0.901** | 1 | 57000 |
| Grizzly | local | NDVI | -0.12 | 0.067 | -0.12 | -0.207, -0.034 | -0.255, 0.009 | 1 | 57000 |
| Grizzly | local | road [high] | -0.144 | 0.212 | -0.146 | -0.415, -0.129 | -0.556, 0.276 | 1 | 23943 |
| Grizzly | local | recreation | 0.256 | 0.129 | 0.255 | **0.092, 0.421** | **0.005, 0.514** | 1 | 57000 |
| Grizzly | local | rec x road | -0.178 | 0.283 | -0.179 | -0.541, 0.183 | -0.733, 0.384 | 1 | 22193 |
| Grizzly | local | rec x logging | 0.073 | 0.294 | 0.074 | -0.299, 0.447 | -0.511, 0.651 | 1 | 36763 |
| Grizzly | landscape | mean rec. | -0.307 | 0.359 | -0.273 | -0.731, 0.077 | -0.113, 0.308 | 1 | 38504 |

**Temporal analysis model estimates**

**Table S3.** Mean posterior estimates and credible intervals (CI) from linear mixed effect models testing the effects of recreation detection rate (per 100 days), disturbance (road density, logging), and sampling effort (number of detections of mule deer, carnivores, or recreation) at camera trap stations on activity overlap between mule deer and carnivores or recreation. Significant covariates are noted in bold. *Det rate* = detection rate. Covariate details are included in the main text as Table 2.

| Comparison | covariate | mean | sd | median | Rhat | n.eff | 80% CI | 95% CI |
| --- | --- | --- | --- | --- | --- | --- | --- | --- |
| Mule deer- recreation | intercept | 0.409 | 0.376 | 1.146 | 1 | 57000 | -0.048, 0.870 | -0.326, 0.408 |
|  | logging[high] | 0.032 | 0.045 | 0.120 | 1 | 39424 | -0.025, 0.089 | -0.056, 0.032 |
|  | mule deer detections | 0.017 | 0.020 | 0.056 | 1 | 20035 | -0.008, 0.042 | -0.022, 0.017 |
|  | rec. detections | 0.017 | 0.019 | 0.055 | 1 | 57000 | -0.007, 0.041 | -0.021, 0.017 |
|  | **road[high]** | **0.131** | **0.042** | **0.214** | **1** | **24868** | **0.078, 0.185** | **0.049, 0.131** |
|  | carnivore det rate | 0.005 | 0.025 | 0.053 | 1 | 57000 | -0.027, 0.036 | -0.044, 0.005 |
|  |  |  |  |  |  |  |  |  |
| Mule deer- carnivores | intercept | 0.614 | 0.322 | 0.613 | 1 | 57000 | -0.181, 0.985 | -0.018, 1.250 |
|  | logging[high] | -0.003 | 0.03 | -0.003 | 1 | 57000 | -0.040, 0.030 | -0.062, 0.056 |
|  | mule deer detections | 0.009 | 0.014 | 0.009 | 1 | 57000 | -0.010, 0.023 | -0.019, 0.037 |
|  | carnivore detections | 0.003 | 0.014 | 0.003 | 1 | 36854 | -0.021, 0.012 | -0.025, 0.030 |
|  | **road[high]** | **0.045** | **0.030** | **0.045** | **1** | **42866** | **0.015, 0.085** | -0.013, 0.104 |
|  | rec det rate | 0.010 | 0.017 | 0.010 | 1 | 41328 | -0.017, 0.024 | -0.024, 0.044 |

**Table S4.** Details regarding hunting pressure for each camera array in our study, based on expert knowledge from Principal Investigators as well as provincial regulations for Wilderness Management Units. Seasonal hunting for focal species is largely concentrated in the fall and winter months (Government of British Columbia 2023; Sports Scene Publications 2023). N/A refers to a species not being present in a given study area. Joffre Lake and Garibaldi Provincial Parks are not shown as no hunting is permitted in either of those parks.

|  |  | | |  | | | |
| --- | --- | --- | --- | --- | --- | --- | --- |
|  | Ungulate | | | Carnivores | | | |
| Camera array | Mule deer | Elk | Moose | Wolf | Grizzly Bear | Black Bear | Cougar |
| Cathedral Prov. Park | Seasonal | Seasonal | Seasonal | Seasonal | Seasonal | Seasonal | Seasonal |
|  |  |  |  |  |  |  |  |
| Golden Ears Prov. Park & Malcom Knapp | Seasonal (Indigenous hunting) | Seasonal (Indigenous hunting) | Not hunted | Not hunted | Not hunted | Not hunted | Not hunted |
|  |  |  |  |  |  |  |  |
| Sea to Sky | Seasonal | Not hunted | Not hunted | Seasonal | Not hunted | Seasonal | Not hunted |
|  |  |  |  |  |  |  |  |
| Kootenays | Seasonal | Seasonal | Seasonal | Seasonal | Not hunted | Seasonal | Seasonal |
|  |  |  |  |  |  |  |  |
| Sooke | Seasonal | N/A | N/A | N/A | N/A | Seasonal | Seasonal |
|  |  |  |  |  |  |  |  |
| South Chilcotins | Seasonal | N/A | Seasonal | Seasonal | Not hunted | Seasonal | Seasonal |
|  |  |  |  |  |  |  |  |
| Yellowhead region | Seasonal | Seasonal | Seasonal | Seasonal | Not hunted | Seasonal | Seasonal |
|  |  |  |  |  |  |  |  |
|  |  |  |  |  |  |  |  |
| Kananaskis | Seasonal | Seasonal | Seasonal | Seasonal | Not hunted | Seasonal | Seasonal |
|  |  |  |  |  |  |  |  |

**References**

Government of British Columbia (2023). Hunting and Trapping Regulations Synopsis. <https://www2.gov.bc.ca/gov/content/sports-culture/recreation/fishing-hunting/hunting/regulations-synopsis>. Accessed July 17 2023.

Sports Scene Publications Inc. (2023) Alberta Guide to Hunting Regulations. <https://albertaregulations.ca/huntingregs/season-wmus.html>. Accessed July 17 2023.

**Supplementary Figures**

**Figure S1.** Distribution of covariates for camera deployments within each camera array; and road density within 500m buffer around each camera station (km/km^2^). Cameras were deployed on linear features (roads or hiking trails). Additional details are provided in Table 2.

**Figure S2**. Detection rate (per 100 days) for focal ungulate species assessed in our study: A) mule deer, B) moose, and C) elk. Rates are from cameras deployed on linear features (roads, hiking trails) across ten projects, 446 camera traps and span the spring and summer months (April to September).

**Figure S3.** Detection rate (per 100 days) for focal carnivore species assessed in our study: A) wolf, B) black bear, C) cougar, and D) grizzly bear. Rates are from cameras deployed on linear features (roads, hiking trails) across ten projects and 446 trap stations. Data shown span the spring and summer months (April to September).

**Figure S4**. Diel activity overlap curve for mule deer and recreation activity, based on detection data pooled across the year.

**Figure S5.** Diel activity overlap curves for mule deer and recreation activity, based on detection data from the A) spring and summer (April to September) and B) fall and winter (October to March) months.
